# Association Between Age of Achieving Gross Motor Development Milestones During Infancy and Body Fat Percentage at 6 to 7 Years of Age

**DOI:** 10.1007/s10995-021-03238-9

**Published:** 2021-10-16

**Authors:** Tomoko Aoyama, Yuki Hikihara, Masashi Watanabe, Hitoshi Wakabayashi, Satoshi Hanawa, Naomi Omi, Hidemi Takimoto, Shigeho Tanaka

**Affiliations:** 1 Department of Nutritional Epidemiology and Shokuiku, National Institutes of Biomedical Innovation, Health and Nutrition, 1-23-1 Toyama, Shinjuku-ku, Tokyo 162-8636 Japan; 2 Japan Society for the Promotion of Science, 5-3-1 Kojimachi, Chiyoda-ku, Tokyo 102-0083 Japan; 3 Faculty of Creative Engineering, Chiba Institute of Technology, 2-1-1 Shibazono, Narashino-shi, Chiba 275-0023 Japan; 4 College of Education, Ibaraki University, 2-1-1 Bunkyo, Mito-shi, Ibaraki 310-8512 Japan; 5 Faculty of Engineering, Hokkaido University, Kita 13, Nishi 8, Kitaku, Sapporo-shi, Hokkaido 060-8628 Japan; 6 Meiji Yasuda Life Foundation of Health and Welfare, 1-8-3, Nishisinjuku, Shinjuku-ku, Tokyo 160-0023 Japan; 7 Faculty of Health and Sport Sciences, University of Tsukuba, 1-1-1 Tennodai, Tukuba-shi, Ibaraki 305-8577 Japan; 8 Department of Nutrition and Metabolism, National Institutes of Biomedical Innovation, Health and Nutrition, 1-23-1 Toyama, Shinjuku-ku, Tokyo 162-8636 Japan; 9 Faculty of Nutrition, Kagawa Nutrition University, 3-9-21 Chiyoda, Sakado-shi, Saitama 350-0288 Japan

**Keywords:** Growth and development, Motor skills, Adiposity, Infant, Child

## Abstract

**Objectives:**

The later achievement of gross motor milestones during infancy is associated with adiposity in early childhood. However, the associations between gross motor development and adiposity after entering primary school are unclear. This study examined the associations between the ages at which six gross motor milestones were achieved and adiposity during early school years.

**Methods:**

This retrospective study was conducted in 2012 and 2013. Data were collected from 225 first-grade primary school children (mean age, 6.9 years; 39% girls). Adiposity was assessed using dual-energy X-ray absorptiometry and expressed as body fat percentage. Data describing the ages of achieving six gross motor milestones (holding head up, sitting, crawling, standing supported, walking supported, and independent walking) were obtained from the Maternal and Child Health Handbooks.

**Results:**

Mean body fat percentage was 21.7%. Multiple linear regression analyses revealed that later ages of achieving crawling (p < .001 [95% confidence interval: 0.33–1.16]), standing supported (p < .001 [95% confidence interval: 0.64–1.65]), and walking supported [p = .013 (95% confidence interval: 0.13–1.07)] were associated with increased fat. However, the ages of achieving holding head up (p = .053), sitting (p = .175), and independent walking (p = .736) were not statistically significant.

**Conclusions:**

Achieving crawling, standing supported, and walking supported later predict increased body fat when aged 6–7 years. The practice of observing gross motor milestone achievements may allow early targeted interventions to optimize body composition before beginning school and thereby, potentially prevent childhood obesity.

## Significance

*What is already known on this subject?* The later achievement of some gross motor milestones in infancy is associated with early childhood adiposity. The association between motor development in infancy and adiposity after entering primary school remains unclear.

*What this study adds?* Later achievements of crawling, standing supported, and walking supported during infancy are associated with adiposity assessed using DXA during early school years. This study demonstrates that the age at which certain gross motor milestones are achieved during infancy may be early predictors of body fat percentage during early school years.

## Introduction

Childhood obesity is now a public health priority (World Health Organization, 2016). Systematic reviews have shown that obesity during childhood tracks to adulthood (Simmonds et al., 2016; Singh et al., 2008), while increasing the risk of both all-cause and cardiovascular disease mortality in later life (Flegal et al., 2013; Prospective Studies Collaboration, 2009). Moreover, research has shown the existence of associations between excess body fat and cardiovascular disease risk factors during childhood (Going et al., 2011). This makes it crucial to identify childhood adiposity at an early time in order to implement effective strategies and interventions designed to prevent obesity across an individual’s life span.

Excess body fat often occurs in conjunction with poor motor skills and/or competencies. In this regard, cross-sectional studies have shown that children of normal weights exhibit better motor competence than those who are overweight or obese (Camargos et al., 2016; Morano et al., 2011); specifically, gross motor skill status (as opposed to fine motor skill status) is associated with body fat in this context (Marmeleira et al., 2017). Previous research has also found associations between body fat and poor motor skills among infants aged 9–24 months (Andres et al., 2013). However, few studies have investigated the longitudinal associations between gross motor development during infancy and adiposity in later life. Shoaibi et al. (2019) reported that later achievement of crawling was associated with higher weight-for-length z scores in male infants aged 12 months (Shoaibi et al., 2019). Benjamin Neelon et al. (2012) found that achieving independent walking later was associated with overall adiposity in children aged three years (Benjamin Neelon et al., 2012). These findings together suggest that later achievements of gross motor milestones predict obesity during early childhood. However, little is known about the associations between gross motor development and adiposity among children after they enter primary school.

Schmidt Morgen et al. (2014) found that the later ages of achievement of sitting and independent walking were associated with lower body mass index (BMI) z scores among children aged seven years, although these associations were of negligible magnitude (Schmidt Morgen et al., 2014). BMI and normative BMI percentiles are often conjunctively used to identify pediatric obesity (Styne et al., 2017), thus, forming a popular index for general adiposity in pediatric research. However, this is not the best measure for distinguishing body fat mass from fat-free mass in children (Wells, 2000). More precise indicators such as body fat percentage (%Fat) estimated using dual-energy X-ray absorptiometry (DXA; Sopher et al., 2004) may provide a clearer understanding of the associations between infant gross motor development and childhood adiposity. This study, therefore, examined whether gross motor development during infancy was associated with adiposity assessed by DXA among children in their early school years.

## Methods

This retrospective study, which used data from Maternal and Child Health Handbooks (MCHHs), took place in the Kanto region, Japan, between 2012 and 2013. Participants included 248 first-grade children (i.e., 153 boys and 95 girls aged 6–7 years) who were recruited from six public schools and two schools affiliated with national universities in Tokyo, Chiba, and Ibaraki prefectures. MCHH data were ultimately collected from 240 participants; of these, 15 were excluded due to very low birth weights (< 1.5 kg) (*n* = 1) or because they were missing all data on gross motor development (*n* = 14). As such, the final sample included 225 children (i.e., 137 boys and 88 girls). This study was approved by the ethics committee at the National Institute of Health and Nutrition in Japan; all participants and their parents were given written explanations of the study procedures. Further, the parents of participants provided written informed consent prior to the participation.

Anthropometric measurements were taken at the National Institute of Health and Nutrition or the University of Tsukuba. Body height and weight were measured to the nearest 0.1 cm and 0.1 kg, respectively; participants only wore light underwear (no shoes). Relative weight (%) was calculated using a formula based on the national reference data for Japanese children (Ministry of Education, Culture, Sports, Science and Technology 2015), which was then classified into three categories: obese (≥ 20%), normal (− 20 to 20%), or thin (≤ − 20%).

$${\text{Relative weight }}\left( \% \right) \, = \, \left[ {{\text{measured weight}}\,\left( {{\text{kg}}} \right)\,\, - \,\,{\text{standard weight}}\,\left( {{\text{kg}}} \right)} \right]/{\text{standard weight }}\left( {{\text{kg}}} \right) \, \times { 1}00$$

$${\text{Standard weight }}\left( {{\text{kg}}} \right) \, = {\text{ a }} \times {\text{ measured height }}\left( {{\text{cm}}} \right) \, - {\text{ b}}$$

a and b are gender- and age-specific values.

BMI was calculated in kg/m^2^ based on height and weight, while %Fat was estimated using DXA (Hologic QDR-4500; Hologic, Inc., Waltham, MA, USA).

Parents transcribed MCHH data to a questionnaire during anthropometric measurements, all of which were subsequently verified by the investigators. Data on maternal factors (height, pre-pregnancy weight, and age at delivery), birth outcomes (gestational age, birth weight, and birth length), and child development during infancy (height, weight, and feeding patterns at health checkups and gross motor development) were obtained through this procedure. Information on maternal smoking before and during pregnancy and the child’s birth order was also obtained thorough the questionnaire. In Japan, MCHHs are designed to keep health records of pregnancy and early childhood (Nakamura, 2010; Takeuchi et al., 2016). They are distributed by local governments when women report their pregnancies. Records on birth and health checkups are recorded by obstetricians, pediatricians, public health nurses, or midwives (Nakamura, 2010). Health checkups are usually performed at hospitals, clinics, or health centers at months 1, 3–4, 6–7, 9–10, 18, and 36. Mothers also use the MCHHs to record their height and pre-pregnancy weight. Further, parents can record information on child development, such as the dates/age at which their children achieve six gross motor milestones, including sitting without support (sitting), hands-and-knees crawling (crawling), standing supported, walking supported, and independent walking. We thus determined the ages (months) at which participants achieved the six gross motor milestones. However, some participants were missing data for these milestones, with 39 having data for all six, 56 having data for five, 46 for four, 31 for three, 21 for two, and 32 for only one. Further, maternal pre-pregnancy BMI and children’s BMI at each health checkup were calculated.

All measured and calculated values were presented as means and standard deviations (SDs). The Student’s t-test was used to investigate potential differences between boys and girls. Partial correlation analyses were used to test the relationships between study variables that were controlled for sex as covariates. Then, multiple linear regression analyses were conducted to determine associations between the ages at which the six motor milestones were achieved and %Fat. We first entered each age of achieving a motor milestone as independent variable and %Fat as a dependent variable (Model 1). We then entered BMI at 3–4 or 9–10 months as an independent variable to examine whether the associations depend on physical growth expressed as BMI during infancy (Model 2). All models were adjusted for sex, birth order, height, maternal pre-pregnancy BMI, gestational age, and school location (prefectures). We also tested the influence of introducing other potential confounders, including maternal age (years) at delivery, maternal smoking, birth weight, feeding patterns at 1 month or 3–4 or 6–7 months, and child age (months) on the model. However, there was no strong evidence of associations between such factors and %Fat, thus, they were excluded from the final model. All statistical analyses were conducted using the IBM®SPSS® software, version 25.0 for Windows (IBM Ltd., Armonk, NY, USA). Statistical significance was set at p < 0.05.

## Results

Of the 225 participants, 1.8% (1 boy and 3 girls) were categorized as obese while none were categorized as thin. Table 1 shows participants’ characteristics. The mean %Fat was 21.7% (SD: 4.5). While boys were generally taller [95% confidence interval (CI): 0.28 to 2.90], %Fat was found to be lower among boys (95% CI: − 4.48 to − 2.20). Boys also achieved standing (95% CI: − 0.77 to − 0.04) and walking (95% CI: − 1.09 to − 0.01) supported at slightly earlier ages than girls. Mothers who gave birth to boys were slightly younger (95% CI: − 2.44 to − 0.002) and had lower pre-pregnancy BMI (95% CI: − 1.54 to − 0.08) when compared to mothers who gave birth to girls. Thus, we included sex as a covariate for the partial correlation analysis.

**Table 1:** Child and maternal characteristics of study participants

Variable	Mean (SD) and range or %			Mean (SD)				p value†
	n	All		n	Boys	n	Girls	
Maternal characteristics								
Pre pregnancy BMI (kg/m^2^)	223	20.3 (2.7)	(16.1–35.4)	136	20.0 (2.3)	87	20.8 (3.2)	**.030**
Age at delivery (years)	225	31.9 (4.6)	(21–44)	137	31.4 (4.2)	88	32.6 (5.0)	**.050**
Maternal smoking (%)	225							
Never smoked	196	87.1						
Smoked before pregnancy	26	11.6						
Smoked during pregnancy	3	1.3						
Child characteristics								
Birth outcomes								
Gestational age (weeks)	225	38.9 (1.5)	(32–41)	137	38.8 (1.5)	88	39.0 (1.4)	.353
Birth weight (g)	225	3009 (408)	(1614–4064)	137	3047 (403)	88	2950 (411)	.080
Birth length (cm)	225	48.9 (2.1)	(43.0–55.0)	137	49.1 (2.1)	88	48.6 (2.1)	.116
Birth order (%)	225							
1	151	67.1						
2	59	26.2						
> 2	15	6.7						
Age of achieving milestones (months)								
Holding head up	133	3.3 (0.6)	(1.4–5.4)	81	3.3 (0.6)	52	3.3 (0.6)	.602
Sitting	106	6.5 (0.8)	(4.8–9.0)	63	6.5 (0.8)	43	6.6 (0.8)	.754
Crawling	159	8.0 (1.5)	(3.8–12.6)	100	8.0 (1.4)	59	8.1 (1.5)	.639
Standing supported	156	8.3 (1.1)	(6.2–12.1)	96	8.1 (1.2)	60	8.5 (1.1)	**.030**
Walking supported	123	9.5 (1.5)	(6.5–13.9)	67	9.2 (1.5)	56	9.8 (1.5)	**.047**
Independent walking	188	12.8 (1.9)	(9–19)	113	12.6 (1.8)	75	13.1 (2.1)	.108
BMI at health checkups (kg/m^2^)								
1 month	220	14.5 (1.3)	(10.2–18.4)	135	14.8 (1.3)	85	14.1 (1.1)	** < .001**
3–4 months	222	17.2 (1.3)	(14.0–21.5)	136	17.3 (1.3)	86	17.0 (1.4)	.069
6–7 months	196	17.3 (1.2)	(13.7–21.1)	118	17.5 (1.1)	78	17.1 (1.3)	**.023**
9–10 months	205	17.1 (1.3)	(13.1–20.0)	126	17.3 (1.2)	79	16.9 (1.3)	**.044**
18 months	222	16.3 (1.1)	(12.9–19.4)	134	16.4 (1.1)	88	16.1 (1.0)	**.010**
36 months	214	15.7 (0.9)	(13.4–18.3)	130	15.8 (1.0)	84	15.6 (0.8)	.327
Feeding patterns at 1 month (%)	210							
Breast feeding	112	53.3						
Mixed feeding	90	42.9						
Formula feeding	8	3.8						
Feeding patterns at 3–4 months	200							
Breast feeding	130	65.0						
Mixed feeding	52	26.0						
Formula feeding	18	9.0						
Feeding patterns at 6–7 months	156							
Breast feeding	95	60.9						
Mixed feeding	35	22.4						
Formula feeding	26	16.7						
Outcome measures at 6 to 7 years								
Age (months)	225	82.9 (3.5)	(76–89)	137	82.8 (3.6)	88	83.0 (3.4)	.758
Weight (kg)	225	21.6 (3.0)	(16.1–33.4)	137	21.9 (3.1)	88	21.1 (2.7)	.068
Height (cm)	225	119.0 (4.9)	(107.1–132.1)	137	119.6 (5.2)	88	118.0 (4.3)	**.017**
BMI (kg/m^2^)	225	15.2 (1.4)	(12.8–22.3)	137	15.2 (1.3)	88	15.1 (1.4)	.645
%Fat (%)	225	21.7 (4.5)	(13.1–37.2)	137	20.4 (4.2)	88	23.8 (4.2)	** < .001**

The data presented are the means and SD (ranges) or proportions (%)

*SD* standard deviations; *BMI* body mass index; *Sitting* sitting without support; *Crawling* hands-and-knees crawling; *%Fat* body fat percentage

†p values were calculated to show differences between sexes using the t-test

Table 2 shows the matrix of partial correlations among early-life factors and childhood measures. As seen, the ages of achieving the six gross motor milestones during infancy showed weak-to-moderate positive correlations with one another (r = 0.26 to 0.74). There were significant positive and weak associations between %Fat at the ages of 6–7 years and the ages of achieving crawling (p < 0.001), standing supported (p < 0.001), and independent walking (p = 0.015). However, holding head up did not reach statistical significance (p = 0.072). Maternal pre-pregnancy BMI (p < 0.001) and child BMI at 3–4, 9–10, 18, and 36 months (p < 0.05) were also positively correlated with %Fat at the ages of 6–7 years. The ages of achieving two milestones (walking supported and independent walking) were positively associated with maternal age at delivery, while the three (holding head up, standing supported, and independent walking) were inversely associated with gestational age. The associations between the ages of achieving the six gross motor milestones and physical growth in terms of BMIs during infancy were largely independent; however, some milestones (sitting, crawling, and independent walking) were negatively associated with BMI at 1 month. In addition, independent walking was associated with BMI at 3–4, 6–7, and 9–10 months. Although not shown in the table, there was a positive correlation between %Fat and BMI at 6–7 years of age (p < 0.001, r = 0.67).

**Table 2:** Partial correlation matrix between early-life factors and childhood adiposity (controlled for sex)

Variables	Age of achieving gross motor milestones (months)						BMI (kg/m^2^)	%Fat (%)
	Holding head up	Sitting	Crawling	Standing supported	Walking supported	Independent walking		
Maternal age at delivery (years)	.037	.164	− .024	.052	.223*	.174*	− .033	.046
Maternal pre-pregnancy BMI (kg/m^2^)	.053	− .022	− .135	− .080	− .081	− .036	.281***	.233***
Gestational age (weeks)	− .222*	− .095	− .127	− .200*	− .079	− .248***	− .079	− .052
Birth weight (g)	− .134	− .133	− .053	− .047	.026	− .100	.111	− .009
Age of achieving gross motor milestones (months)								
Holding head up		.467***	.405***	.282**	.258*	.290**	.109	.157
Sitting			.288**	.355***	.382**	.346**	.144	.090
Crawling				.740***	.618***	.429***	.048	.263***
Standing supported					.706***	.473***	.064	.298***
Walking supported						.456***	.008	.221*
Independent walking							− .007	.048
BMI at health checkups (kg/m^2^)								
1 month	− .169	− .271**	− .158*	.020	.018	− .160*	.052	− .003
3–4 month	− .021	− .102	.011	.011	.079	− .146*	.181**	.164*
6–7 months	.027	.052	.034	− .049	.019	− .177*	.147*	.097
9–10 months	.100	.031	.037	.005	.127	− .161*	.303***	.211**
18 months	.131	.079	.093	.129	.158	.109	.342***	.142*
36 months	.112	.041	.014	− .002	− .026	.038	.559***	.285***

*BMI* body mass index; *Sitting* sitting without support; *Crawling* hands-and-knees crawling; *%Fat* body fat percentage

*p < .05

**p < .01

***p < .001

Table 3 shows the results of the multiple linear regression analyses on the associations between the six gross motor milestones and %Fat (Model 1). The ages at which the participants achieved crawling (β = 0.23), standing supported (β = 0.29), and walking supported (β = 0.20) were significantly and positively associated with %Fat. These associations were still significant after adjusting for the BMI at 9–10 months (Model 2). The age of holding head up was significant (β = 0.15) only when the BMI at 3–4 months was adjusted (Model 2).

**Table 3:** Multiple linear regression analyses on the associations between gross motor milestones and adiposity

Age of achieving gross motor milestones (months)	n	%Fat (%)			
		B	β	p value	95% CI
Model 1					
Holding head up	133	1.05	.14	.053	**− **0.01–2.12
Sitting	105	0.64	.12	.175	**− **0.29–1.56
Crawling	158	0.75	.23	** < .001**	0.33–1.16
Standing supported	154	1.15	.29	** < .001**	0.64–1.65
Walking supported	121	0.60	.20	**.013**	0.13–1.07
Independent walking	186	**− **0.05	**− **.02	.736	**− **0.36–0.26
Model 2					
Holding head up	132	1.11	.15	**.043**	0.04–2.18
Sitting	104	0.73	.13	.119	**− **0.19–1.65
Crawling	145	0.75	.23	** < .001**	0.32–1.18
Standing supported	143	1.21	.30	** < .001**	0.69–1.73
Walking supported	111	0.55	.19	**.022**	0.08–1.02
Independent walking	167	**− **0.10	**− **.04	.567	**− **0.43–0.23

Model 1: Adjusted for sex, height, birth order, maternal pre-pregnancy BMI, gestational age, and school location

Model 2: Same as Model 1 plus BMI at 3–4 months for holding head up and sitting, BMI at 9–10 months for crawling, standing supported, walking supported, and independent walking

*B* non-standardized regression coefficient; *β* standardized regression coefficient; *CI* confidence interval; *Sitting* sitting without support; *Crawling* hands-and-knees crawling; *%Fat* body fat percentage; *BMI* body mass index

## Discussion

This study retrospectively investigated the associations between adiposity during early school years and the ages at which six gross motor milestones were achieved during infancy. Results showed significant associations between the later ages of achieving three milestones and increased body fat among participants at the ages of 6–7 years. These associations were not dependent on maternal pre-pregnancy BMI, gestational age, and BMI at 9–10 months. Each 1-month increase in the age of achieving standing supported was associated with 1.2% increase in body fat at 6–7 years of age, even when considering the BMI at 9–10 months (Model 2). Given that the ages at which children first stand with support are distributed over a period of almost six months (Table 1), it can explain body fat differences by a maximum of nearly 7% (approximately 1.5SD unit in this cohort). However, these associations were weak and therefore, their clinical significance must be carefully examined.

The ages of achieving the gross motor milestones assessed in this study, except holding head-up, are comparable to the “windows of achievement” for motor milestone among healthy children (WHO Multicentre Growth Reference Study Group, 2006). Most data were within the windows, however, there were four cases where the milestones were achieved earlier than the lower limit (2 for crawling and 2 for walking supported) and three cases where they were achieved later than the upper limit (1 for standing supported and 2 for independent walking). Removing these cases did not affect the significance of the results, however, the magnitude of β changed slightly (data not shown). These outliers are probably due to the subjective reports provided by the parents rather than objective assessments, however, the ages reported as achieving later than the windows were all within one month of the upper limits, suggesting that our study cohort’s gross motor development was not extremely delayed and was essentially normal. Additionally, the prevalence of obesity was 1.8%, which is lower than the average (4.1%) in Japanese six-year-old children, reported in the 2013 school health survey (Ministry of Education, Culture, Sports, Science and Technology 2013), demonstrating that our study cohort may have a smaller range of body shape and be healthier than the population. These findings suggest that age differences within the normal range for achieving the three milestones during their infancy may determine the percentage of body fat in healthy children during their early school years.

To our knowledge, this is the first study to examine the associations between the ages at which gross motor milestones were achieved and adiposity assessed by DXA in later life. Previously, Benjamin Neelon et al. (2012) examined the achievement of four gross motor milestones and skinfold thickness among children aged three years, while Schmidt Morgen et al. (2014) examined the achievement of two gross motor milestones and BMI among children aged seven years. Although anthropometric measurements such as BMI (Wells, 2000) and skinfold thickness (Noradilah et al., 2016) are used as proxies for body fat, BMI cannot distinguish fat mass from fat-free mass and skinfold thickness does not refer to fat-free mass. Therefore, BMI may be suboptimal for researching childhood obesity. Indeed, there was only a moderate correlation (r = 0.67) between %Fat and BMI at 6–7 years of age. Further, BMI at 6–7 years of age could not be used to determine significant associations with the ages at which motor milestones were achieved (Table 2). In addition, skinfold thickness does not necessarily reflect a constant proportion of total body fat and is associated with technical errors during the measurement process. However, DXA is known as a reliable and valid method for objectively assessing whole-body fat mass and fat-free mass (Sopher et al., 2004). Therfore, this study’s results provide robust evidence supporting associations between later achievement of certain gross motor milestones and subsequent adiposity. This indicates that the implications of infant motor development may persist longer regarding later adiposity, than previously thought (Benjamin Neelon et al., 2012).

Although there are consistent positive intercorrelations among the ages at which children achieve the six gross motor milestones, this study only found significant associations between %Fat and crawling, standing supported, and walking supported (Model 1). Andres et al. (2013) found associations between poor motor skills and %Fat among infants aged 9–24 months, but not for infants under 6 months (Andres et al., 2013), suggesting that motor development during the first 6 months is less likely to reflect later adiposity. This may explain the current lack of strong evidence about the associations between adiposity and the achievements of holding head up and sitting. However, the achievement of independent walking was not significantly associated with adiposity, although this only develops after 9 months of age. Further, this does not support Benjamin Neelon et al.’s study (2012). These discrepancies may partly be related to the reporting forms used for the MCHHs; that is, while the achievement of independent walking was recorded by age (years and months), the other milestones were recorded by date (month and day). Thus, the age at which independent walking was achieved was rather crude, which may have masked its actual association with %Fat.

Since gross motor activities such as crawling, standing supported, and walking supported expend more energy to maintain posture and/or locomotion, the age at which these milestones are achieved may represent daily physical activity during infancy. Achieving gross motor milestones at later ages were associated with lower levels of physical activity in infants and toddlers (Hnatiuk et al., 2013; Prioreschi et al., 2017). Additionally, physical activity was associated with central adiposity in infants (Benjamin Neelon et al., 2020). Therefore, achieving these milestones later is likely to be associated with physical inactivity during preschool years and may, thereby, cause subsequent body fat accumulation. Another possibility is that %Fat during infancy determines the timing of certain gross motor developments and is carried over to the early school years. During infancy, gross motor milestones are achieved when infants are able to apply the amount of muscular strength that is needed to control their bodies (Malina 2004). Since children with excess body fat require more strength to support themselves and/or move, more time may be needed to achieve each gross motor milestone. Indeed, greater body fat percentages are associated with lower psychomotor developmental index scores in infants aged 9–24 months (Andres et al., 2013); this excess body fat will persist from infancy to toddler age (Forsum et al., 2019) and then to childhood (Blair et al., 2007). These rationales are not contradictory and can happen in an individual.

Gross motor development is a multifactorial process in which various factors are involved. A systematic review looked into the factors associated with gross motor development in infants from birth to achieving independent walking; two child-related factors (birth weight and gestational age) and one environmental factor (sleeping position) have been identified to be associated with gross motor development (Boonzaaijer et al., 2021). More importantly, randomized control trials have shown that nutritional (Torsvik et al., 2015) and exercise (Zelazo et al., 1972) interventions may accelerate gross motor development. However, it is currently unclear whether these interventions that facilitate gross motor development have long-lasting benefits on the body composition. This would be worth studying in the future.

This study has several limitations. First, we obtained data on the dates/age at which six gross motor milestones were achieved from MCHH records. Since these records were based on subjective reports provided by parents rather than on objective assessments, they may have been biased, which could have affected the results. Parents may have reported that their children achieved the milestones earlier than the actual date/age, because medical staff can check MCHHs during infant health checkups and some parents may not want to be told that their child is delayed. Additionally, each parent’s interpretation of achieving a milestone is different since “achievement” is not well defined in the MCHH. Second, since the data were recorded at the parents’ discretion, there were a considerable number of cases with missing data. This also forms another unknown bias—which may have affected the results. Additionally, it prevented the evaluation of sequences or specific patterns related to milestone achievement. Particularly, crawling is not necessarily observed in all children (4% were identified as non-crawlers) and it is not observed in any specific order (42% crawl before standing supported, 36% crawl after standing supported, and the others have different patterns; WHO Multicentre Growth Reference Study Group, 2006). Additional research is needed to examine these points further. Third, this retrospective study used a small sample. Therefore, large cohort studies that follow children from birth are needed to establish associations between infant motor development and increased body fat at later stages. Although caution should be used when interpreting findings due to these limitations, this study also has several strengths. For instance, it precisely measured adiposity using DXA, the results of which provide a better understanding of the early developmental origins of adiposity.

In conclusion, this study found that the later achievements of crawling, standing supported, and walking supported were early predictors of adiposity among first-grade children. Although these associations were weakly significant, they were observed over a longer period (i.e. beyond beginning school) compared to previously studies. Gross motor development is universal; further, it is easy to identify the ages at which gross motor milestones are achieved without equipment. This will help the caretakers understand the child’s future risk of adiposity, even without information regarding their height or weight. Therefore, it seems practical to observe gross motor milestone achievement to target infants who may need early interventions to prevent childhood adiposity. This will provide opportunities to optimize their body composition earlier in life. However, future studies should continue examining the associations between infant gross motor development and later adiposity in other populations.

## Data Availability

Not applicable.
